# DipSkmer: Reference-free population genomics with diploid genome skims

**DOI:** 10.64898/2026.06.05.730460

**Published:** 2026-06-07

**Authors:** Eduardo Charvel, Homère J. Alves Monteiro, Siavash Mirarab, Vineet Bafna

**Affiliations:** 1 Bioinformatics and Systems Biology Graduate Program, UC San Diego, La Jolla, California, USA; 2 Center for Evolutionary Hologenomics, GLOBE Institute, University of Copenhagen, Copenhagen, Denmark; 3 Department of Electrical and Computer Engineering, UC San Diego, La Jolla, California, USA; 4 Department of Computer Science & Engineering, UC San Diego, La Jolla, California, USA

## Abstract

Ecologists and conservation biologists rely on genetic diversity as a key essential biodiversity variable (EBV) used to track population health and dynamics, and utilize the population parameter *θ* (estimated by the average pairwise genomic distance) as a key metric of diversity. While whole-genome-sequencing (wgs) is increasingly affordable, it will be considerable time before the full diversity of life is represented by high-quality assembled genomes; even then, constant monitoring will still require repeated sampling of populations. In contrast, genome skimming (low-coverage, short-read wgs) is highly cost-effective but challenging to analyze because the coverage is too low for assembly and reliable error correction. Mature methods, such as Mash, exist for estimating pairwise genomic distances based on the Jaccard similarity of *k*-mer sets computed using sketching techniques. Some, such as Skmer, additionally model the impacts of low coverage. These methods have been successfully applied to assembly-free species identification and phylogenetics; however, their use in population genetics has been limited. This is because these methods implicitly treat genomes as haploid and heterozygosity confounds true estimates of genomic distance for diploid organisms. In this paper, we address this problem through a number of technical advances. First, we use coalescent theory to mathematically derive how the Jaccard index between two diploid samples changes with the scaled population size parameter (*θ*). Next, we derive an estimator that computes *θ* from the Jaccard index, in addition to several auxiliary variables, which we also estimate from the genome skims. The resulting method, DipSkmer, enables more accurate estimates of coverage, sequencing error, and pairwise nucleotide distance for diploid samples. Analyses of both simulated and empirical datasets show that for diploids and low distances (e.g., < 2%), DipSkmer produces the most accurate pairwise distance estimates, outperforming existing alignment-free methods such as Mash and Skmer, and closely approximates ANGSD, a reference and alignment-based tool.

**Availability:**

The code for DipSkmer is available at https://github.com/echarvel3/ReSkmer/tree/DipSkmer-REFACTOR. Simulation scripts and environments are available at https://github.com/echarvel3/dipskmer_scripts.

## Author Summary

The process of obtaining full-genome population genomic measurements for biodiversity monitoring remains expensive due to the need for high-coverage sequencing and reference assemblies. Genome skimming has been shown to be a viable, low-coverage alternative for obtaining genomic distances, and alignment- and assembly-free methods exist for analyzing nuclear data from skimming data to estimate the distance between samples. However, existing methods fail to model within-sample heterozygosity, expected for diploid organisms. Given the dominance of diploidy among species of interest to ecologists, the implications of these simplifying assumptions warrant further study. Here, we present a mathematical model of the *k*-mer sets sampled from two diploid genomes from a Wright-Fisher population. We use the model to develop DipSkmer, a *k*-mer-based, reference-free method for estimating nucleotide diversity and population divergence that, unlike its predecessors, models within-sample heterozygosity. Benchmarking shows more accurate genomic diversity estimates compared to existing reference-free, genome-skimming methods and comparable performance to the popular high-coverage, reference-based method, ANGSD. Thus, DipSkmer enables accessible, less expensive population monitoring through genetic diversity estimates.

## 1 Introduction

Nucleotide diversity, measured as the percentage of positions in the genome that differ between two organisms, is widely used across biology. This distance measure is used in phylogenetics, microbiome analyses, comparative genomics, and population genetics as a simple measure of evolutionary divergence. In particular, nucleotide diversity is among the Essential Biodiversity Variables (EBV) used extensively by ecologists and conservation biologists to monitor the health of species^35^. Species with dwindling populations suffer from reduced genetic diversity^16^, which can lead to loss of fitness^41^. Monitoring the health of species, which is increasingly important as biodiversity continues to face unprecedented levels of strain^8,17^, requires not just a one-time estimate of diversity but continuous monitoring across spatial and temporal gradients. Such a goal can only be achieved if we have access to low-cost tools for monitoring.

When reference genomes and highly covered samples are available, nucleotide diversity can be measured by calling genotypes and computing the Hamming distance. Under the Wright-Fisher (WF) model, Hamming distance corresponds^29^ to *θ* = 2*μN_e_* for haploid and *θ* = 4*μN_e_* for diploid populations, where *μ* is the substitution rate per nucleotide per site, and *N_e_*, the effective population size, is what interests the ecologists. However, much of the biodiversity on earth is not yet represented with reference genomes, and conservation biologists cannot afford to wait until such sampling is done. Even when reference genomes are available, high-coverage samples are expensive to obtain if repeated sampling is required. The viable alternative of barcoding a single marker gene gives limited resolution at the species level^25,37^.

An alternative that has been put forward^4^ and used with some success^2,7,9,10,13,24,28,52^ is genome skimming, which consists of sequencing a sample at low coverage without assembling the nuclear genome (mitogenome can be assembled, a method many skimming-based analyses use^12^). Skimming can have a low cost and can eliminate the need for assembly. The *k*-mer sketching methods can then be used to efficiently compute distances between sets of reads for species identification. In particular, Ondov et al.^34^ developed an efficient approach to use min-hash^5^ to estimate the Jaccard (*J*) index between the *k*-mer sets of two samples, and used a simple transformation noted earlier by Fan et al.^14^ to translate the Jaccard index to nucleotide distances. Later, Sarmashghi et al.^43^ showed how the translation between *J* and distance can also account for effects of low coverage and sequencing error by pairing it with an analysis of the *k*-mer frequency spectrum, and Charvel et al.^11^ showed how to account for the impact of repeats. Many researchers have built on various hashing techniques for fast distance calculation^1,18,22,36,40,45^.

Despite this rich literature, existing methods have largely overlooked the complexity introduced by within-sample heterozygosity. These methods fail to account for changes between the two alleles present in a single sample of a diploid organism, and instead model evolutionary changes on one chromosome, effectively assuming haploidy. Such a model is suitable for application to microbial species, but insufficient for diploid organisms. Ignoring within-sample heterozygosity may be inconsequential when comparing samples from different species (e.g., for species identification), but it is important for population genetic analyses at low distances. The fundamental unanswered mathematical questions are: Given reads generated from two diploid individuals sampled from a Wright Fisher evolving population with scaled population size θ = 4μN_e_, how does the Jaccard index between their k-mer sets relate to θ, and how does Jaccard change as coverage drops and sequencing errors are added? We resolve these questions here.

First, we use coalescence theory to characterize the Jaccard index with respect to *θ* for diploid genomes (Methods 4.2). Next, we show how Jaccard changes given low-coverage genome skims (Methods 4.3), and how to estimate parameters needed for Jaccard computation (Methods 4.4). We use these derivations to develop DipSkmer (Diploid Skmer), which takes as input two sets of reads from closely related diploid organisms and outputs their nucleotide distance. In extensive simulations, DipSkmer shows substantially higher accuracy compared to Mash and Skmer for diploid samples. On real data, we show that DipSkmer’s *θ* estimates correlate strongly with those from high-coverage data mapped to a reference genome.

## 2 Results

### 2.1 Diploidy Alters the Jaccard Index’s Relationship with Nucleotide Diversity

**Figure 1:**
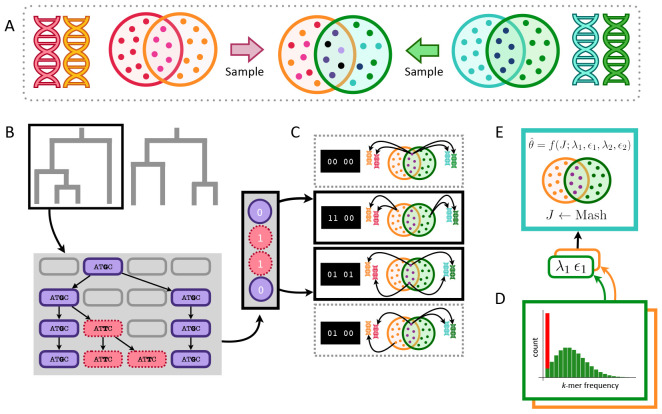
DipSkmer estimates nucleotide diversity between two diploid genomes, using only low coverage, error-prone, unassembled genomic data. **A)** The Jaccard index computed from genomes of two diploid organisms measures similarity between two pairs of haploid chromosomes, where each sample contains its own homozygous and heterozygous *k*-mers. **B)** We can model the possible coalescence scenarios of the four independent *k*-mers obtained from each position of two diploid genomes. **C)** For each topology, depending on the branches where mutations are introduced, *k*-mers may appear in one or both samples with computable probabilities (see Fig. 6). **D)** The alleles resulting from these mutations are then independently sorted into each haploid, leading to 0, 1, or 2 shared *k*-mers per position. **E)** We incorporate a model to estimate coverage (*ξ*) and error (*ϵ*) based on *k*-mer frequency spectrum of the sample; erroneous *k*-mers (red) tend to be less frequent, whereas error-free *k*-mers tend to follow a Poisson distribution (green). **F)** We derive the expected numbers of *k*-mers shared in scenarios shown in (D) to design an estimator of nucleotide diversity (Eqs. (2) and (3)) given an observed Jaccard index and estimates of coverage and error.

When comparing *k*-mers between two diploid genome skimming samples, each sample carries its own set of heterozygous and homozygous *k*-mers, which are in turn homozygous or heterozygous across both samples, thereby creating a complex relationship between within-sample heterozygosity and *k*-mer set similarity (Fig. 1A). At the individual *k*-mer level, it is hard to know whether it is heterozygous in each sample. However, by assuming the Wright-Fisher model, we can model the expected number of *k*-mers following particular evolutionary patterns, and thereby characterize how genetic diversity shapes the Jaccard Index. In DipSkmer (Diploid Skmer), we use a coalescent model to capture how *k*-mers, treated as independent haplotype blocks, can have distinct evolutionary histories (Fig. 1B-C). Given the resulting *k*-mer alleles, we then model how they are represented in the Jaccard Index (Fig. 1D), and finally incorporate a coverage and error model to account for missing and erroneous *k*-mers within the diploid framework (Fig. 1E), yielding a final estimate of *θ* (Fig. 1F).

#### Estimating *θ* between diploid samples.

Under a diploid model, *k*-mer loci will occur twice in a DNA sample, making it so that each locus can weight the Jaccard differently with different configurations of heterozygous k-mers (Fig. 1D). DipSkmer’s nucleotide diversity model (Methods: Extending to two diploid individuals (*n* = 4)) assumes that all haplotypes are sampled from a randomly mating population, such that coalescence between the paternal and maternal alleles within a sample is identically distributed to coalescence between alleles from different individuals. Each set of haplotypes can then be traced back to one of two topologies, each with its own probability of occurrence (Fig. 1B). Under the infinite sites assumption, we model how mutations may fall on each topology (Fig. 1C) and assign a random permutation of haplotypes to the topology leaves (Fig. 1D). We then analyze the expected number of *k*-mers in the intersection (*I*) and union (*U*) at a single genomic position. Since different substitution patterns contribute differently to the union and intersection, enumerating all scenarios yields: $E[I]=1+\frac{1}{11}(1-Q)$ and E[U]=2−Q. Using the method of ratios from Mash/Skmer and solving for *Q*, we obtain:

J=1+∕111(1−Q)2−Q⇒Q=2J−∕11121+J−∕1112.

#### Diploid parameter estimation.

To the diploid Jaccard model, we introduce a low-coverage model (Methods: Modeling low coverage and errors) that estimates the probability of a locus contributing an observed *k*-mer to the union and intersection of the Jaccard Index, given estimates of *k*-mer coverage (*λ*) and sequencing error rate (*ϵ*) (Fig. 1E). To estimate these sequencing parameters, we derive a new set of equations that account for how within-sample heterozygous *k*-mers affect the distribution of observed *k*-mer counts (Methods: Parameter estimation for diploid skims). Specifically, we take the ratio of consecutive *k*-mer count bins to obtain an error-free *k*-mer coverage estimate (*ξ*), which yields an internal, per-sample estimate of *θ* and, in turn, a *θ*-informed estimate of *λ* and sequencing error rate *ϵ*.

### 2.2 Simulation Results

In order to test DipSkmer’s accuracy, we use msprime^3^ to simulate diploid populations of different base genomes as as explicitly defined in Experimental setup. By modulating effective population size, simulated samples result in having different nucleotide diversity values (*θ* ϵ {0.001, 0.003, 0.006, 0.009, 0.012}). To test whether DipSkmer can also estimate the divergence between populations, we perform separate msprime simulations that lead to populations having different levels of genetic divergence (0.03125, 0.0625, 0.125, 0.25, 1) in coalescent units (*τ*/4*N_e_*). Note that these simulations violate the random mating assumptions of DipSkmer, and thus, test the robustness of the method (especially at higher divergence levels). Moreover, the presence of repeated *k*-mers violates DipSkmer assumptions; we focus on three base genomes with *k*-mer uniqueness above 0.7 in the main results. To test the degradation of performance with extremely high levels of repeats, we separately examine three extra genomes (Experimental setup). After generating genomes, we use ART^15^ to simulate reads for each simulation at varying coverages (*c* ϵ {0.5×, 1×, 2×, 4×, 8×}).

We compare DipSkmer to its predecessor alignment-free methods, Skmer (which effectively assumes haploid input) and Mash (which additionally ignores low coverage). We also compare to ReSkmer, which like Skmer ignores heterozygosity but unlike DipSkmer models repeats in the genome. We compare *θ* estimates using these methods to the measure of true *θ* (the percentage of substituted positions in genome). We measure error as $\left(\hat{\theta }-\theta \right)∕\theta$, averaged over all replicates, where $\hat{\theta }$ is the estimated pairwise distance and *θ* is the true pairwise distance. We also report mean absolute error (MAE), defined as $|\hat{\theta }-\theta |∕\theta$. For population differentiation experiments, we estimate *D_xy_* (average *θ* between pairs of samples taken from different populations) and Weir and Cockerham’s $F_{st}=1-\frac{\theta_{x}+\theta_{y}}{2D_{xy}}$^48^, comparing estimated values to the ground truth, calculated by counting substitutions.

#### 2.2.1 DipSkmer provides better *θ* estimates with multiple iterations

Before comparing DipSkmer to other methods, we first compare per-sample and per-pair estimates of *θ*. Recall that we use *k*-mer frequency spectrum to obtain a *θ* for each sample used in coverage calculations (Section 4.4), in addition to the pairwise estimate of *θ* based on Jaccard. In simulated datasets, pairwise Jaccard-based estimates of *θ* are far more accurate than within-sample frequency-spectrum-based estimates, especially with low coverage (Fig. S2). Based on this observation, we propose an iterative approach to estimate *θ* using the within-sample method, estimate coverage, estimate pairwise *θ*, and use this new *θ* to obtain better coverage, iterating this process several times. DipSkmer’s average error reduces from 46% with the within-sample method to 29% error in the third pass of the iterative approach (Fig. S3). With the exception of high coverages (4× and 8×), the third iteration of DipSkmer is more accurate than using within-sample estimates from Equation (7). On empirical data, correlations stay stable across passes (Fig. S4). However, going from one pass to three passes tends to help accuracy in cases where the true *θ* is much lower than the starting θ* used in the first pass (0.005). While it is not universally the best, the third pass is used in all subsequent DipSkmer results.

##### 2.2.2 DipSkmer *θ* estimates are more accurate than haploid methods

**Figure 2:**
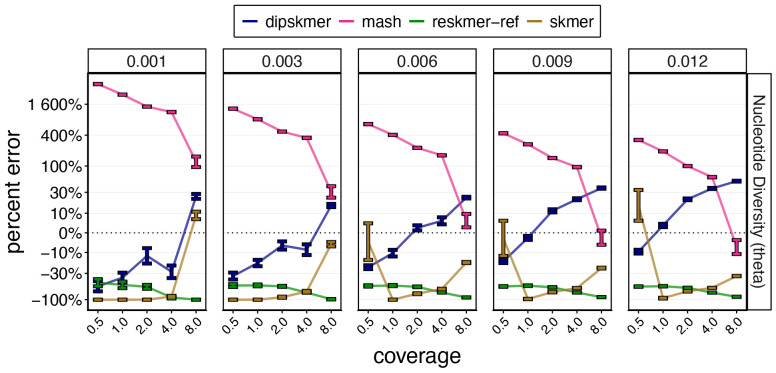
Accuracy of Nucleotide Diversity (*θ*) Estimates on Simulated Data. Percent error ($\left(\hat{\theta }-\theta \right)∕\theta$) plotted against sequencing coverage. Panels correspond to the true *θ* value for each simulated population size. Bars show the range of error across 5 replicates of three genomes. *Note*: error of −100% means the method estimated $\hat{\theta }=0$.

Across most coverages, populations, and parental genomes, DipSkmer provides substantial improvements compared to ReSkmer-ref, Skmer, and Mash (Fig. 2). Overall, DipSkmer has the smallest MAE (25%), which is a substantial improvement compared to ReSkmer (67%), Skmer (86%) and Mash (619%) (Fig. S5). Comparing coverage levels, DipSkmer obtains its highest accuracy at 2× (with 17% MAE) where it provides substantial improvements over ReSkmer-ref (57%), Skmer (81%) and Mash (476%). Only at 8×, the highest simulated coverage, does Skmer achieve comparable accuracy (19% MAE) to DipSkmer. Mash has the best accuracy at 8× coverage when *θ* is ≥ 0.009, but is otherwise inaccurate; it has very high error when coverage is below 4× or when estimating small distances (*θ* < 0.005); even at 8× coverage, it has an error of 190% for these small distances.

When comparing methods at very low coverages (0.5× and 1×) DipSkmer is far more accurate than other methods. At these coverages, Skmer consistently outputs an estimate of zero when the simulated *θ* ≤ 0.003. ReSkmer-ref is as accurate as DipSkmer for *θ* ≤ 0.003, but not for higher *θ*. Examining each of the three parental genomes separately (Figs. S5 and S6), we observe that DipSkmer is especially accurate in the least repetitive genome we simulate (leech) with a MAE of 20% (compared to ReSkmer-ref’s 79%, Skmer’s 70%, and Mash’s 626%). DipSkmer’s error for the two more repetitive genomes only increases to 28%, while Skmer error rises to 97%. Among these moderately repetitive genomes (UR>0.7), ReSkmer-ref becomes more accurate as repeat content increases, but remains less accurate than DipSkmer in most situations. However, for extremely repetitive genomes (*UR* ϵ {0.6; 0.5; 0.4}) and low *θ* (*θ* = 0.001 – 0.003), ReSkmer-ref becomes more accurate than DipSkmer (Fig. S7), which often produces 0 distance. Even for these genomes, DipSkmer is competitive with ReSkmer at higher *θ* values, outperforming ReSkmer at low coverages.

We also test how accurately DipSkmer estimates sequencing parameters compared to Skmer and ReSkmer (Fig. S8). In terms of coverage, error rate, and genome length, DipSkmer clearly outperforms Skmer. There is a large improvement in sequencing error estimates at 0.5× and 1× coverage, with more modest gains for higher coverages. The estimated coverage and genome length similarly improve the most at lower coverages but gain very little at higher coverages. ReSkmer-ref understandably outperforms DipSkmer in genome length and coverage estimations, given we are providing an error-free reference genome. In spite of not being reference-based, DipSkmer obtains more accurate sequencing error estimates than ReSkmer-ref when coverage ≥ 2×. Despite these improvements, which are likely due to modeling within-sample *θ* in repeat-spectrum analyses, some overestimation of coverage and thus underestimation of genome length remain, perhaps due to inaccuracies in estimating within-sample *θ* (Fig. S2).

##### 2.2.3 DipSkmer accurately measures population divergence with *D_xy_* and *F_ST_*

**Figure 3:**
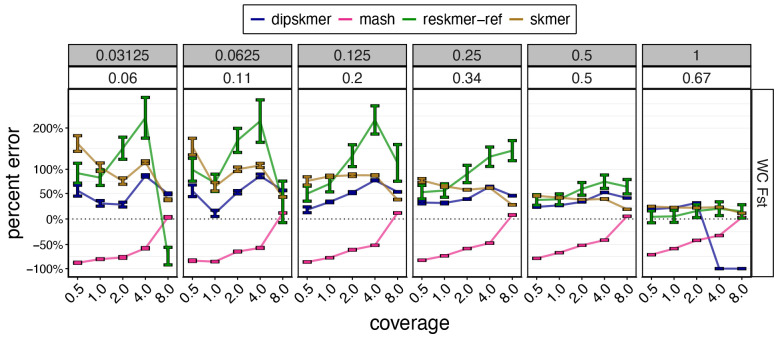
Accuracy of fixation index (*F_st_*) on simulated data. We show results for Weir and Cockerham *F_st_* across two simulated populations at a constant population size (*N_e_* = 5 × 10^6^). Panels correspond to different levels of divergence in coalescent units (gray strip) with the corresponding mean true WC *F_st_* shown below.

We next measure DipSkmer’s ability to measure divergence between populations in simulated data, starting with the fixation index, Weir and Cockerham *F_ST_* (Fig. 3). DipSkmer and Skmer both overestimate *F_ST_*, but DipSkmer has a lower error in most conditions, with some exceptions among high coverage and high divergences (coalescent unit 1 divergence, which often corresponds two different species). Notably, with lower coverage, DipSkmer is much more accurate than Skmer, and its accuracy is highest at around 1–2× coverage. Mash has the opposite error, underestimating *F_st_* in all cases with coverage below 8×. Mash essentially fails to detect differentiation between populations when coverage is below 4×. Conversely, Mash is very accurate when the population differentiation is at the speciation level (1 coalescent unit) and coverage is high (i.e., conditions it is designed for); in these conditions, which strongly break DipSkmer’s assumptions of random coalescence, DipSkmer fails to detect differentiation at 4–8× coverage. Nevertheless, DipSkmer produces accurate estimates at 0.5–2× coverage despite strong model misspecification. We also test two more definitions of *F_ST_*, Nei’s and Slatkin’s, and observe similar results (Fig. S9). Repeats play a role in all methods’ *F_st_* estimation accuracy (Fig. S10); DipSkmer achieves its highest accuracy in low repeat genomes and becomes less accurate in higher repeat genomes. However, the relative accuracy of methods remained similar across repeat levels.

Another measure of divergence we test is the average distance between samples of different populations, *D_xy_* (Fig. S11). For *D_xy_*, DipSkmer is the most accurate method in most conditions, with some exceptions when divergence is very high or the coverage is 8×. Skmer and Mash both perform better than DipSkmer at 8× coverages. ReSkmer-ref has a similar performance to Skmer, but becomes more inaccurate as coverage increases. The *D_xy_* metric clearly shows the impact of population divergence on DipSkmer: As population divergence increases (breaking random coalescence assumptions), the accuracy of *D_xy_* declines steadily, especially above 0.25 coalescent units divergence. In contrast to *F_st_* and *θ*, repetitiveness does not affect *D_xy_* estimation accuracy much. DipSkmer’s accuracy stays around 31%-32% regardless of repeat level (Fig. S11).

### 2.3 Biological Results

To test DipSkmer on empirical data, since true *θ* values are not known, we compare *k*-mer-based methods to previously published^26^ *θ* estimates obtained by ANGSD^21^, which is a widely used method based on read mapping, genotyping, and computing Site Frequency Spectrum (SFS). The dataset is composed of 6 species and 12 populations (Table S2). To get measures of correctness of our nucleotide diversity estimates, we measure the difference between *θ* values and the Pearson correlation of our estimates of *θ* to those produced by ANGSD. We follow a published protocol^27^ for the preparation of reads (described in Methods). We then subsample all genome skims to target coverages *c* ϵ {0.5×, 1×, 2×, 4×, 6×, 8×}.

#### 2.3.1 DipSkmer closely matches ANGSD *θ* estimates in empirical datasets

**Figure 4:**
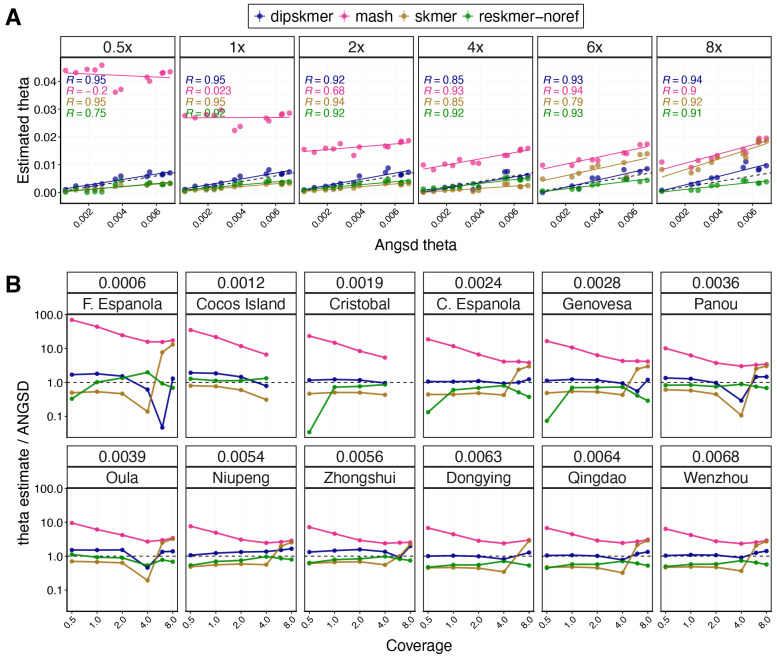
Correlations and Accuracy in Empirical Datasets. **A)** Correlation of DipSkmer, Skmer, and Mash estimates of *θ* (*y*-axis) to ANGSD (*x*-axis). Each panel corresponds to a different level of coverage. Pearson correlation coefficients (R) are found in the top left corner of each panel. **B)** Ratio (*y*-axis) between DipSkmer, Skmer, and Mash *θ* estimates and ANGSD *θ* across different coverages (*x*-axis). Panels represent different populations, with population name and corresponding ANGSD estimate on the top strips.

On the biological dataset, to gauge DipSkmer’s ability to quantify relative changes in nucleotide diversity, we first test how well DipSkmer correlates with ANGSD estimates of *θ* (Fig. 4A). According to the Pearson correlation coefficient (*R*), DipSkmer has the highest correlation averaged across coverages (*R*=0.92), followed closely by Skmer (*R*=0.9) and ReSkmer-noref (*R*=0.9), all of which perform far better than Mash (R=0.43). Mash is essentially uncorrelated at lower coverages, starts to have some correlation at 2×, and matches other methods at 6× or higher. DipSkmer, in contrast, has high correlation coefficients at all coverage levels, with minimal change across coverage.

Beyond correlations, DipSkmer also closely matches ANGSD estimates of *θ*, in contrast to Mash, which systematically overestimates, and Skmer, which systematically underestimates ANGSD (Fig. 4B). ReSkmer-noref is competitive with DipSkmer, except at 0.5× coverage, where ReSkmer underestimates *θ* in some populations (see C. Espanola, Cristobal, and Genovesa). Across all populations and coverage, DipSkmer produces the most similar distances to ANGSD judged by MAE (28%) compared to ReSkmer-noref (41%), Skmer (83%), and Mash (738%); this pattern remains correct for each coverage level (Table S1). Overall, the impact of coverage on DipSkmer is relatively low, with MAE ranging between 22% and 40%, with the peak accuracy achieved at 2× coverage. Meanwhile, ReSkmer-noref achieves its peak accuracy at 4× coverage (30% MAE), where it is nevertheless outperformed by DipSkmer (26%).

For Sillago Sinica populations (Dongying, Qingdao, and Wenzhou) we test the similarity of reference-free estimates of *θ* between pairs of samples to those produced by an ANGSD-produced identity by state (IBS) matrix. IBS measures a normalized genetic distance between aligned loci given reference genomes and high coverage samples – which DipSkmer does not require. Again, DipSkmer produces the most similar estimates to ANGSD (Fig. S12), with a mean percent difference of 12% across all coverages. The two next most accurate methods have similar percent error: ReSkmer-noref (30%) and Skmer (43%). There is no substantial change in error between coverages, except for Mash whose error decreases from 687% (0.5×) to 237% (6×). Notably, even though coverage is relatively high, Mash remains highly inaccurate.

#### 2.3.2 DipSkmer best approximates ANGSD estimates of *Sillago sinica* population structure

To test how DipSkmer performs in the presence of population structure, we take three populations from the same species (*Sillago sinica*) and look at two metrics for comparison: *F_st_* and Principal Coordinates Analysis (PCoA). We perform PCoA on identity by state (IBS) matrices produced by ANGSD as well as those produced by reference-free methods to project distances onto Euclidean space, followed by Procrustes to align the projections to enable comparison across methods.

**Figure 5:**
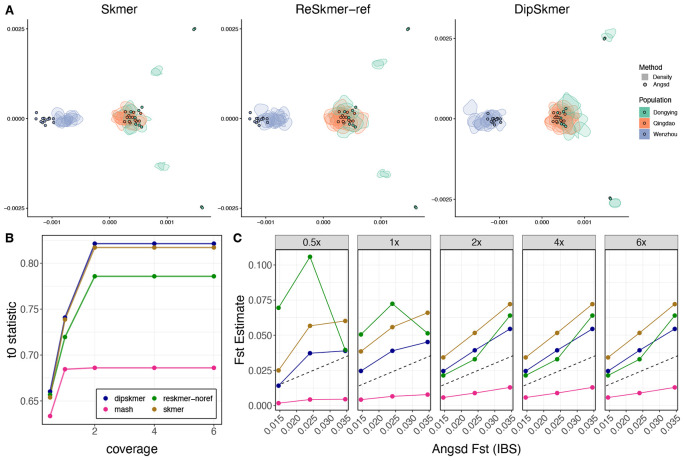
*Sillago sinica* Population Structure: **A)** Similarity between PCoA produced by ANGSD and those produced by three different alignment-free methods. Colors represent three different *Sillago sinica* populations. Points show the coordinates of ANGSD PCoA results and contours show the location of the highest 33% density of data points for each sample using the corresponding method. **B)** Similarity in PCoA structure (*t*_0_) between PCoA produced by ANGSD and those produced by skimming methods. The *t*_0_ statistic ranges from 0 (dissimilar) to 1 (identical). **C)** Correlation plot between reference-free estimates of Weir and Cockerham *F_st_* (*y*-axis) and those produced using the ANGSD IBS matrix (*x-axis*). The dotted line denotes the optimal relationship between estimates.

Projections based on DipSkmer distances are remarkably close to those based on ANGSD (Fig. 5A). In fact, in most cases, ANGSD projections are within the confidence intervals for DipSkmer (obtained using subsampling reads, see Methods). Skmer and ReSkmer-ref also capture the broad patterns but show lower similarity in their projections to those of ANGSD, in particular for four outlier Dongying samples. Judged by the alignment of points to ANGSD, as measured by the *t*_0_ similarity score (see Methods), DipSkmer is closest to ANGSD across all coverages and achieves very high similarity (*t*_0_ = 0.821) for 2× – 6× (Fig. 5B). All methods have lower *t*_0_ values at coverage ≤ 1×, reach their peak at 2×, with little change at higher coverages.

To test the accuracy of *F_st_* measurements across methods, we use the distances output by reference-free methods to compute Weir and Cockerham (WC) *F_st_* (see Methods) and compare those to WC *F_st_* calculated from ANGSD IBS matrices (Fig. 5C). At 0.5× and 1×, DipSkmer produces the most similar estimates to ANGSD. However, at > 1×, ReSkmer-noref produces slightly more similar estimates to ANGSD for two population pairs, Qingdao-Dongying and Wenzhou-Qingdao, followed by DipSkmer. Finally, we also compare our *F_st_* values to those produced by 2D-SFS analysis with ANGSD (Fig. S13). Here, again, DipSkmer is the most accurate among reference-free methods at low coverages (22% error when coverage < 2×).

## 3 Discussion

Here we presented DipSkmer, a method that models diploidy’s effect on the Jaccard index of *k*-mer sets to produce accurate and robust estimates of nucleotide diversity *θ* from diploid samples. In both simulated and empirical datasets, DipSkmer outperformed other alignment-free, reference-free methods for diploid inputs. Additionally, it was able to estimate measures of population differentiation such as *F_st_* and *D_xy_* with high accuracy. Moreover, DipSkmer is fast, requiring only 5 minute on average on our datasets for each sample (Table S4), which is identical to Skmer, and not much higher than Mash, which needs one minute per sample.

The DipSkmer’s promise for practical biodiversity monitoring lies in providing estimates that highly correlate with the more laborious option of mapping reads to a reference genome (ANGSD). Crucially, since DipSkmer eliminates the need for access to reference genomes, it opens up the possibility for population monitoring using genome skims for less well-studied species. And the fact that it requires low coverage reduces the cost. In fact, DipSkmer was generally more accurate with 2× coverage than 4×, which may seem counterintuitive. We refer to Remark S1 (in supplementary material) for some theoretical insights as to why this may be the case: Around 4× coverage, changing *θ* surprisingly has little to no effect on how many *k*-mers are shared between two samples, reducing sensitivity. In practice, when coverage is 3–5×, we suggest downsampling reads to 2×.

The theory we provide allows estimation of *θ* from a single sample using its *k*-mer repeat spectrum or using two samples by modeling Jaccard. Given the ability to estimate heterozygosity from a single sample, which others have also attempted^19^, one may wonder why the Jaccard-based estimates were necessary. Our empirical results showed that within-sample estimates of *θ* are far less robust, especially with low-coverage data (Fig. S2). In contrast, with correct coalescent modeling, Jaccard distances between pairs of samples provide highly accurate estimates of *θ*. Thus, we advise practitioners to use the pairwise estimates rather than single-sample estimates. Pairwise estimates additionally enable comparing populations (e.g., *D_xy_* and *F_st_*), which is not possible with single-sample *k*-mer statistics.

While our theory was based on Wright Fisher (WF) populations with random mating, DipSkmer was empirically robust when applied to individuals from two divergent populations, including at very low coverage and low population divergence. In extreme simulated cases where population separation approached the speciation-level divergences (e.g., around 1 coalescence unit in simulations), the accuracy of DipSkmer distance estimates drops. Thus, the use of this model should clearly be restricted to comparisons within a species or very close species. When populations are highly diverged, the impacts of within-sample heterozygosity become negligible compared to phylogenetic divergence, making other methods such as ReSkmer, Skmer, or Mash more appropriate. Despite our empirical robustness to moderate levels of population structure, future research should explore how differing coalescence dynamics in demographic models with population isolation and migration^31,49^ affect the Jaccard index in theory.

Just as DipSkmer is an extension of Skmer that models heterozygosity, the recent ReSkmer method is an extension that models repeats based on *k*-mer frequency spectrum, which can be computed using Respect^44^. Unlike DipSkmer, ReSkmer (and Respect) ignore the impacts of heterozygosity. One should ideally model both aspects, but analytical equations become intractable. While we leave it to future work to combine the two models, we can empirically ask which aspect has a higher impact – accounting for repeats or heterozygosity. Based on our results, overall, heterozygosity appears to have a greater impact on Jaccard than repeats for population genetic level distances, though this pattern is not universal. When repeat content is extremely high and one has access to accurate reference genomes to compute *k*-mer frequency spectrum, ReSkmer can be more accurate (Fig. S7). Our results motivate future attempts to combine repeat modeling with within-sample heterozygosity. Due to the complexity of the resulting equations, the combined analyses may require moving beyond analytical approaches and adopting machine learning, following some earlier attempts^47^. Such combined modeling may also enable us to move beyond diploid organisms and model polyploidy and mixed-ploidy.

## 4 Methods

### 4.1 Background: Jaccard index for two haploids within a coalescence framework

The known connections between Jaccard and *θ* can be arrived at by analyzing the coalescent on *n* = 2 haploid samples from a WF population. Let each *k*-mer be considered a set of *k* independent characters. For haploids, the expected time to coalesce under the WF model is $E[t_{2}]=N_{e}$, and thus, the expected number of mutations on the lineage is Λ := 2*N_e_μk* = *θk*. The number of mutations can be considered a Poisson process with parameter Λ; then, $Q≔\text{Pr}[\text{no mutation on the lineage}]=e^{-\Lambda }≃{\left(1-\theta \right)}^{k}$. Thus, for each position of genomes of length *L*, with probability *Q*, a single *k*-mer is added to both the intersection (*I*) and the union (*U*) of *k*-mer sets. If there is at least a mutation (probability 1 − *Q*), the position adds 0 *k*-mers to the intersection and 2 *k*-mers to the union. Thus, E[I]=QL and E[U]=L(2(1−Q)+Q). Using the (biased) ratio estimator, one can arrive at $J=\frac{Q}{2(1-Q)+Q}=\frac{Q}{2-Q}$, which, solved for *θ* would give^14,34^ $\hat{\theta }=1-{\left(\frac{2J}{1+J}\right)}^{1∕k}$.

These calculations fail at low coverage and ignore sequencing errors^43^. Define *L* := length of haploid genome, *N* := number of sampled base pairs, ℓ := read length, $\lambda ≔\frac{N}{L}\frac{\ell -k+1}{\ell }$ *k*-mer coverage. We assume each *k*-mer has probability $\rho ≔{\left(1-\epsilon \right)}^{k}≃e^{-k\epsilon }$ of being error-free and let *ξ* := *λρ* be the error-free coverage of *k*-mers. Sarmashghi et al.^43^ showed that *ϵ*, *λ* can be estimated for each sample from their *k*-mer frequency profile. Assuming random errors, *η* := Pr[an arbitrary *k*-mer at a specific location is sampled without error] = 1 − *e*^−ξ^. Assuming errors generate unique *k*-mers, ζ≔E[the number of uniquek-mers generated from a position]=η+λ−ξ, where *η* captures the error-free *k*-mers and the other terms the erroneous ones. Sarmashghi et al.^43^ showed that with those estimates at hand for both samples (denoted by subscript), the hamming distance (thus, *θ*) can be estimated using

(1) $$1-{\left(\frac{2(\zeta_{1}L_{1}+\zeta_{2}L_{2})J}{\eta_{1}\eta_{2}(L_{1}+L_{2})(1+J)}\right)}^{1∕k}.$$

### 4.2 Extending to two diploid individuals (*n* = 4)

We now turn to the diploid case, where we assume *n* = 4 haploids are sampled from a randomly mating population, so that coalescence between parental and maternal alleles of a sample is identically distributed to alleles from different individuals. We start by analyzing genomic *k*-mers (i.e., all *k*-mers are sampled without error), then model the impacts of low coverage and sequencing errors. Recall that for diploids, *θ* := 4*N_e_μ*.

**Figure 6:**
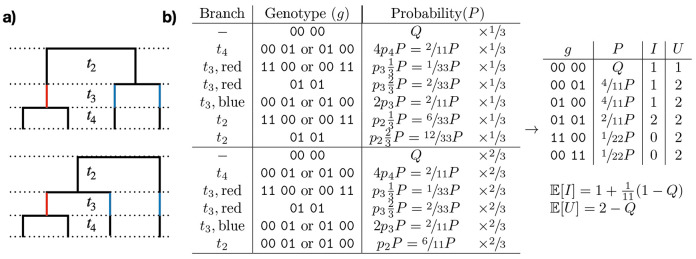
a) Possible coalescence topology shapes. Assignment of alleles to leaves is uniformly random. The epochs *t*_2_ … *t*_4_ are marked. Note that in *t*_3_, the red branch can coalesce with either blue ones to give the unbalanced tree, and thus, the probability of the unbalanced tree is ⅔ versus ⅓ for balanced. b) If a substitution occurs (probability *P* = 1 − *Q*), depending on which branch it falls on, it leads to different genotypes. Each genotype contributes 0–2 *k*-mers to the intersection (*I*) and 1 or 2 *k*-mers to the union *U* of the *k*-mer sets obtained from two diploid genomes.

Consider the coalescence history of each locus, which can take one of two shapes (Fig. 6a), with the unbalanced case twice as likely as the balanced. Under Kingman’s coalescence^20^, for each time epochs $t_{i}\;(i\in \{2,3,4\})$ with *i* lineages, $E[t_{i}]=\frac{2N_{e}}{i\left(i-1\right)∕2}$. The expected total branch length is $E[\sum_{i=2}^{4}it_{i}]=\sum_{i=2}^{4}\frac{4N_{e}}{i(i-1)}i=\frac{11}{6}4N_{e}$. Under the infinite sites assumption, the number of substitutions per *k*-mer falling on these genealogies is Poisson distributed with parameter $\Lambda ≔\frac{11}{6}4N_{e}\mu k=\theta \frac{11}{6}k$ and $Q≔\text{Pr}[\text{no mutation in lineage of}\;n=4\;\text{leaves}]=e^{-\Lambda }≃{\left(1-\theta \right)}^{\frac{11}{6}k}$ (compare to haploids). With probability *P* := 1 − *Q*, we have one or more substitutions in a *k*-mer. We only analyze the possibility of zero or one substitutions, essentially assuming that in the unlikely event that multiple substitutions occur, the ratio of the expected number of shared *k*-mers to all *k*-mers is well approximated by derivations for one substitution. Conditioned on having exactly one substitution, $p_{i}≔\text{Pr}[\text{substitution falls on a specific lineage of epoch}\;t_{i}]=\frac{t_{i}}{\sum_{i}it_{i}}$, and thus, p2=∕113, p3=∕111, p4=∕221.

We assign a random permutation of haplotypes to the leaves of the topologies and analyze the expected number of *k*-mers in the intersection (*I*) and union (*U*) for a single genomic position. Figure 6B summarizes analyses of both topologies. With probability *Q*, no substitutions occur, which leads to *I* = 1, *U* = 1. If any mutation occurs, *U* = 2. If it falls on any lineage in *t*_4_, the genotypes of the two individuals is some permutation of 00 01, which means, *I* = 1. If the mutation falls on the first lineage in *t*_3_, the two lineages carrying the mutation may belong to the same individual (this happens in 8 out of 24 = ⅓ of all permutations), resulting in *I* = 0, or they may belong to different individuals, leading to *I* = 2. If the mutation falls on other lineages of *t*_3_, then *I* = 1. The *t*_2_ epoch depends on the topology, where the balanced tree has the same scenarios as *t*_3_, leading to *I* = 0 or (twice as likely) *I* = 2. For unbalanced, regardless of the permutation, both samples will have one of each allele, leading to *I* = 2. Listing all scenarios, we conclude: $E[I]=1+\frac{1}{11}(1-Q)$ and E[U]=2−Q. Using the same method of ratios as Mash/Skmer and solving for *Q*, we obtain:

J=1+∕111(1−Q)2−Q⇒Q=2J−∕11121+J−∕1112

which adds −∕1112 to numerator and denominator of the haploid equation. Setting $Q=e^{-\Lambda }≃{\left(1-\theta \right)}^{\frac{11}{6}k}$ gives:

(2) θ^=−6111klog(2J−∕11121+J−∕1112)≃1−(2J−∕11121+J−∕1112)6111k

This diploid estimate of $\hat{\theta }$ gives a higher value than the haploid estimate (Fig. S1A) and is only defined for J>∕116. To see why, note that under the infinitive sites assumption (which breaks at higher distances), two samples with high *θ* would still have substantial similarity because the two alleles of each individual are sampled from the same pool of alleles available in the population. As shown in Section SA.2.2, a haploid estimate based on Jaccard can be transformed to the diploid estimate using a non-linear equation.

### 4.3 Modeling low coverage and errors

We next study Jaccard for genome skims where some *k*-mers are not covered and others are erroneous. Recall definitions of *L*, *N*, ℓ, *ρ*, *ξ* from Section 4.1. We henceforth update the definition of $\lambda ≔\frac{1}{2}\frac{N}{L}\frac{\ell -k+1}{\ell }$ to be the *haploid k*-mer coverage for a diploid skim, and note that this is half of what we defined before. We aim to model the fact that due to sequencing errors and limited sequencing coverage, a *k*-mer could be missing and/or sampled erroneously in one or both samples, thereby reducing the intersection and potentially increasing the union. Mash deals with errors by requiring each *k*-mer to be observed at least *t* times, noting that for a threshold *t* sufficiently large compared to *λ*, a *k*-mer sampled *t* times is likely to be error-free. This approach only works for high coverage, since for low coverage, very few *k*-mers will survive this filter. Our approach is to select a *t* ≥ 1 based on our estimate of coverage. In practice, we set t=⌊∕2ξ⌋+1 by default, allowing each sample *j* to have its own *t_j_*. We assume that no erroneous *k*-mer will match any alleles in the genome. Let

$$\psi_{j}≔E[\text{number of erroneous kmers per position appearing at least}\;t_{j}\;\text{times in sample}\;j].$$

Thus, each position in expectation adds *ψ*_1_ + *ψ*_2_ erroneous *k*-mers to the union and none to the intersection. Since errors are mostly random, with our choice of *t_j_*, it is safe to assume *ψ*_*j*_ = 0 for *t_j_* > 1 and *ψ*_*j*_ = 2*λ*(1 − *ρ*) for *t_j_* = 1. We now focus on the error-free *k*-mers, which may be homozygous or heterozygous. Let

$$\eta_{j}(i)≔\text{Pr}[\text{sample}\;j\;\text{includes at least}\;t_{j}\;\text{copies of a}\;k\text{-mer present}\;i\;\text{times in the genome}]=1-F_{P}(t_{j}-1;i\xi )$$

where *F*_P_ is the CDF of Poisson, which can be calculated using the regularized incomplete gamma function. Since a *k*-mer may be homozygous or heterozygous (effectively covered twice), we are interested in *i* ϵ {1, 2}. For each location, and for each genotype (Fig. 6), we can compute their expected contribution to *U* and *I*, as listed below, from which E[I]=P⋅I and $E[U]=P\cdot U+\psi_{1}+\psi_{2}$ can be calculated (see Eqs. (S5) and (S6)).

**Table:** 

Genotype g	P	I=E[I∣g]	U=E[U∣g,error-free]
00 00	Q	$\eta_{1}(2)\eta_{2}(2)$	$\eta_{1}(2)+\eta_{2}(2)-\eta_{1}(2)\eta_{2}(2)$
00 01	$\frac{4P}{11}$	$\eta_{1}(2)\eta_{2}(1)$	$\eta_{1}(2)+\eta_{2}(1)-\eta_{1}(2)\eta_{2}(1)+\eta_{2}(1)$
10 00	$\frac{4P}{11}$	$\eta_{1}(1)\eta_{2}(2)$	$\eta_{1}(1)+\eta_{2}(2)-\eta_{1}(1)\eta_{2}(2)+\eta_{1}(1)$
01 01	$\frac{2P}{11}$	$2\eta_{1}(1)\eta_{2}(1)$	$2(\eta_{1}(1)+\eta_{2}(1)-\eta_{1}(1)\eta_{2}(1))$
11 00 or 00 11	$\frac{P}{11}$	0	$\eta_{1}(2)+\eta_{2}(2)$

Note: SD - standard deviation; NA - not applicable

For example, for the genotype 00 01, *I* = 1 if and only if the *k*-mer is sampled at least *t*_1_ times from the first skim where it is homozygous, and *t*_2_ from the second skim, where it only appears in one chromosome; otherwise, *I* = 0. Therefore, $E[I|00\;01]=\eta_{1}(2)\eta_{2}(1)$. For union *U*, we have two distinct *k*-mers. Allele 0 is sampled at least *t*_1_ and *t*_2_ times without error with probabilities *η*_1_ (2) and *η*_2_(1) in each individual and *η*_1_(2)*η*_2_(1) in both, and allele 1 with probability *η*_2_(1) in the second individual. Thus, $E[U|00\;01]=\eta_{1}(2)+\eta_{2}(1)-\eta_{1}(2)\eta_{2}(1)+\eta_{2}(1)$. Other scenarios are similar. Replacing E[I] and E[U] from Equations (S5) and (S6) in $J=\frac{E[I]}{E[U]}$ and solving for Q (ratio estimator) results in:

(3) $$Q=1+11\frac{J(\eta_{1}(2)+\eta_{2}(2))-(1+J)\eta_{1}(2)\eta_{2}(2)+\psi_{1}+\psi_{2}}{6J(2\eta_{1}(1)+2\eta_{2}(1)-\eta_{2}(2)-\eta_{1}(2))+(1+J)(-4(\eta_{1}(1)\eta_{2}(1)+\eta_{1}(1)\eta_{2}(2)+\eta_{1}(2)\eta_{2}(1))+11\eta_{2}(2)\eta_{1}(2))}.$$

### 4.4 Parameter estimation for diploid skims

To compute Eq. (3), we need to estimate parameters *λ*, *ϵ* which immediately give us *ξ*, *η*, *ψ*. Following Skmer, we compute these from the *k*-mer repeat spectrum of each sample, but we model heterozygosity. Let *M_i_* denote the number of *k*-mers observed exactly *i* times and assume all errors fall in *M_1_*. Let

$$\;ℳ≔\text{the set of all unique kmers in a diploid genome of length}\;L\;A_{x,i}≔\text{the event that a genomic kmer}x\text{appears}i\text{times error-free in a samplet}.$$

Let M≔∣ℳ∣ and note M≥L and $E[M]=L(2-{\left(1-\theta \right)}^{k})$ (Section SA.1). Clearly, we need to estimate *θ* for each sample in order to estimate genome length and coverage. Let As shown in Eq. (S1), for *i* > 1,

(4) $$E[M_{i}]=\text{Pr}(A_{x,i}|x\in ℳ)=\frac{e^{-2\xi }\xi^{i}((2^{i}-2e^{\xi }){\left(1-\theta \right)}^{k}+2e^{\xi })}{i!(2-{\left(1-\theta \right)}^{k})}.$$

A family of equations can be obtained by taking the ratio of two consecutive instances of Eq. (4) for *i* ≥ 2:

(5) $$\nu_{i}=\frac{E[M_{i+1}]}{E[M_{i}]}=\frac{\text{Pr}(A_{x,i+1}|x\in ℳ)}{\text{Pr}(A_{x,i}|x\in ℳ)}=\frac{2\xi }{\left(i+1\right)}\left(\frac{e^{\xi }-(e^{\xi }-2^{i})\;{\left(1-\theta \right)}^{k}}{2e^{\xi }+(2^{i}-2e^{\xi })\;{\left(1-\theta \right)}^{k}}\right).$$

Using $\nu_{i+1}$ and $\nu_{i}$ enables us to completely eliminate *θ* (see Eq. (S3)) and allows us to arrive at an estimate of *ξ*:

(6) ξ^=∕43(i+1)νi−∕41νi(i+1)(9(i+1)νi−8(i+2)νi+1)

With $\hat{\xi }$ estimated, we then use Eq. (5) to estimate *θ* directly. It can be confirmed that

(7) $$\hat{\theta }=1-{\left(\frac{2e^{\xi }(\xi -\nu_{i}(1+i))}{\nu_{i}(i+i)(2^{i}+2e^{\xi })+2\xi (e^{\xi }-2^{i})}\right)}^{1∕k}$$

satisfies (5), giving us an estimator for *θ*. While any *i* can be used, we follow Skmer^43^ and set it to arg ${\text{arg max}}_{i>1}$ ${\hat{M}}_{i}$ (i.e., the first mode of the skim’s *k*-mer repeat spectrum).

Note that when $9(i+1)\nu_{i}<8(i+2)\nu_{i+1}$, Eq. (6) has no solutions, and a different strategy must be used. Let us assume a θ* is given. Then, we can use Eq. (5) directly to estimate *ξ* by numerically optimizing ${\text{arg min}}_{\xi }{\left(\nu_{i}-\frac{2\xi }{1+i}(\frac{e^{\xi }-(e^{\xi }-2^{i}){\left(1-\theta^{*}\right)}^{k}}{2e^{\xi }+(2^{i}-2e^{\xi }){\left(1-\theta^{*}\right)}^{k}})\right)}^{2}$. For θ*, we can start with a fixed value (default: θ* = 0.005). When multiple individuals are available, we can then use an iterative approach to update θ*. In each pass *r*, we use the pairwise sample distances from the previous round (i.e., $\theta_{r-1}$, as described in Section 4.3) as the new $\theta_{r}^{*}$ to recompute coverage and error, which we then use to obtain a new pairwise distance $\theta_{r}$. We continue for a fixed number of passes (default: three), and empirically compare this method to using Equation (7).

Regardless of which method is used to compute $\hat{\xi }$, we next need to decompose it into *λ* and $\rho ={\left(1-\epsilon \right)}^{k}$. To do so, we cannot use the family of equations given by (5) because they are only a function of *λ*. Instead, we need to examine error-prone *k*-mers with *i* = 1. Note that $E[\sum_{i=1}^{\infty }iM_{i}]=2L\lambda =2\lambda \frac{E[M]}{2-{\left(1-\theta \right)}^{k}}$.

Then, since all erroneous *k*-mers are assumed to be in *M*_1_, as shown in Eq. (S4), we can drive:

E[Mi]={E[M]e−2ξξi((2i−2eξ)(1−θ)k+2eξ)i!(2−(1−θ)k)i≥2E[M]2(λ−e−2ξ(eξ−1)ξ((1−θ)k+eξ)2−(1−θ)ki=1}⇒λ^=E[M1][Mi]12i!ξi((2ie−2ξ−2e−ξ)(1−θ)k+2e−ξ)+ξ(eξ−1)(e−2ξ(1−θ)k+e−ξ)

for any *i* > 1. We can then estimate the error rate from $\hat{\lambda }$ and $\hat{\xi }$ as $\hat{\epsilon }=1-{\left(\hat{\xi }∕\hat{\lambda }\right)}^{1∕k}$.

### 4.5 Experimental setup

#### Simulations.

To test the accuracy of DipSkmer, we used the coalescent simulator msprime^3^ to generate four haploid chromosomes with a range of *θ* values. To achieve this, we set rates of mutation (*μ*) and recombination (*r*) to 0.3 × 10^−8^ (which is reasonable^32^), and vary effective population sizes (*N_e_* ϵ {8.3 × 10^4^, 2.5 × 10^5^, 5 × 10^5^, 7.5 × 10^5^, 1 × 10^6^}), which lead to *θ* = 4*N_e_μ* to be 0.001, 0.003, 0.006, 0.009, or 0.012, resp. We repeat the process 5 times for three genome sizes (15 replicates in total). For each replicate, we divide the four chromosomes into two diplid individuals at random. To test whether DipSkmer can also estimate the divergence between populations, we perform separate msprime simulations with a demography where an initial parent population (*N_e_* = 5 × 10^5^) splits into two child populations with no change in *N_e_* and no migration. Two diploid samples are generated for each of the child populations (eight chromosomes in total). To test different levels of divergence, we adjust the number of generations (τ) back in time when the populations split ($\tau \in \{6.25\times 10^{4},1.25\times 10^{5},2.5\times 10^{5},5\times 10^{5},1\times 10^{6}\}$). These lead to coalescent unit ($\tau ∕4N_{e}$) divergences of {0.03125, 0.0625, 0.125, 0.25, 1}. Note that 1 coalescent unit is high and corresponds to the phylogenetic (rather than population genetic) scale; it is included to test the limits of the methods. Also note that the isolation between populations breaks the random mating assumptions of DipSkmer, and thus, this experiment is meant to test the impacts of model violations.

We generate genomes and genome skims for each simulation. To make these realistic in terms of *k*-mer repeat spectra, we replace the parental genome (root) with one of three assemblies with varying *k*-mer repetitiveness (Table S3). We impose the genotypes (i.e., VCFs) generated by msprime onto the corresponding sites of the genome, selecting among alternative nucleotides at random and leaving the unmutated sites unchanged. For each resulting genome, we use ART^15^ to simulate Illumina short reads (Phred score = 25, ℓ = 150) at coverage *c* ϵ {0.5×, 1×, 2×, 4×, 8×}, noting that each of the two diploid chromosomes has a coverage of ∕2c. We use the resulting reads as input to DipSkmer, with 1 − 3 passes of θ* calculations, to DipSkmer with Eq. (5) for coverage, and alternative Jaccard-based methods, Mash^34^ and Skmer^43^. Variants of DipSkmer differ in the calculation of within-sample *θ* used to estimate coverage; however, the accuracy is always measured based on the *θ* obtained from pairs of samples. We measure error as $\left(\hat{\theta }-\theta \right)∕\theta$, averaged over all replicates, where $\hat{\theta }$ is the estimated pairwise distance and *θ* is the true pairwise distance. We also report mean absolute error (MAE), defined as $|\hat{\theta }-\theta |∕\theta$. We measure the true distance as the percentage of substituted positions in the simulated genome. For population differentiation experiments, we estimate Dxy (average heterozygosity between pairs of samples taken from different populations) and Weir and Cockerham’s $F_{st}=1-\frac{\theta_{x}+\theta_{y}}{2D_{xy}}$^48^, comparing estimated values to the ground truth, calculated based on simulated genomes.

#### Biological data.

To test DipSkmer on empirical data, since true *θ* values are not known, we compare DipSkmer, Skmer, and Mash to previously published^26^ *θ* estimates obtained by ANGSD^21^, which is a widely used method based on read mapping, genotyping, and computing Site Frequency Spectrum (SFS). We analyze a dataset^26^ consisting of 12 total populations across six different species with a range of *θ* between 0.0006 – 0.0068 (Table S2). Beyond the difference between *θ* values, we also measure the Pearson correlation of our estimates of *θ* to those produced by ANGSD. To reduce the impacts of contamination on alignment-free methods^38^, we utilize a published protocol^27^, which includes the removal of adapters and deduplication of reads using BBtools^6^. Bacterial and archaeal reads are then removed using CONSULT-II^54^, and human reads are filtered out using Kraken2^50^. To test methods with low-coverage skims, we use Respect^44^ to estimate genome size, then subsample the coverage of all genome skims to target coverages *c* ϵ {0.5×, 1×, 2×, 4×, 6×, 8×}. Note that two populations (Cocos island and Cristobal) were not sequenced deep enough to reach 8× coverage and are absent from high coverage Pearson correlations.

To benchmark DipSkmer’s ability to measure population structure in an empirical dataset, we analyze a short-read WGS dataset of three *Sillago sinica* populations (Wenzhou, Qingdao, Dongying)^53^. To prepare the sequencing reads for ANGSD^21^, we first performed adapter and quality trimming using BBtools^6^ and used BWA-MEM^23^ to map reads to a publicly available reference assembly^51^. We then use ANGSD to obtain an IBS (identity by state) matrix, run a principle coordinate analysis (PCoA) on it, and analyze the first two components. To judge the ability of reference-free methods to obtain population structure, we first used Skmer’s built-in subsampling approach^39^ to generate 100 replicates of reads subsampled from 4× genome skims. We then use Skmer, ReSkmer, and DipSkmer, to construct distance matrices based on those subsamples. Next, we use the Procrustes method to align each of these distance matrices to ANGSD’s PCoA and summarize the results by plotting polygons in regions with 33% highest density of points per sample (representing confidence intervals). To quantify the similarity between ANGSD and other methods at a range of coverages, we use the vegan package^33^ in R to perform a symmetric Procrustes analysis repeatedly (a statistical test referred to as “protest”) to obtain *t*_0_, a statistic derived from the sum of squares (*t*_0_ = 1 − *ss*) between rotated matrices. This tells us how similar projections from reference-free methods are to those produced by ANGSD; *t*_0_ ranges from 0 (dissimilar) to 1 (identical).

To get *F_st_* values with the reference-free methods, we take the distance matrices output by each method and compute Weir and Cockerham’s (WC) definition of *F_st_*^48^ ($F_{st}=1-\frac{\theta_{x}+\theta_{y}}{2D_{xy}}$). To get *F_st_* with ANGSD, we use two different approaches. First, we take the distances from the IBS matrix to compute WC *F_st_* (referred to as “ANGSD *F_st_* (IBS)”). Second, we use ANGSD and realSFS to generate the 2D Site Frequency Spectra (2D SFS) between all pairs of populations and obtain the unweighted Reynold’s *F_st_*^42^.

## Supplementary Material

Supplement 1

## References

[1] Baker Daniel N. and Langmead Ben. Dashing: fast and accurate genomic distances with HyperLogLog. Genome Biology, 20(1):265, December 2019. ISSN 1474-760X. doi: 10.1186/s13059-019-1875-0. URL https://genomebiology.biomedcentral.com/articles/10.1186/s13059-019-1875-0. 31801633 PMC6892282

[2] Bandaranayake Pradeepa C. G., Naranpanawa Nathasha, Bhagya Chandrasekara C. H. W. M. R., Samarakoon Hiruna, Lokuge S., Jayasundara S., Bandaranayake Asitha U., Pushpakumara D. K. N. G., and Wijesundara D. Siril A.. Chloroplast genome, nuclear ITS regions, mitogenome regions, and Skmer analysis resolved the genetic relationship among Cinnamomum species in Sri Lanka. PLOS ONE, 18(9):e0291763, September 2023. ISSN 1932-6203. doi: 10.1371/journal.pone.0291763. URL https://dx.plos.org/10.1371/journal.pone.0291763. 37729154 PMC10511092

[3] Baumdicker Franz, Bisschop Gertjan, Goldstein Daniel, Gower Graham, Ragsdale Aaron P, Tsambos Georgia, Zhu Sha, Eldon Bjarki, Ellerman E Castedo, Galloway Jared G, Efficient ancestry and mutation simulation with msprime 1.0. Genetics, 220(3):iyab229, 2022. 34897427 10.1093/genetics/iyab229 PMC9176297

[4] Bohmann Kristine, Mirarab Siavash, Bafna Vineet, and Thomas M. Gilbert P.. Beyond DNA barcoding: The unrealized potential of genome skim data in sample identification. Molecular Ecology, 29(14): 2521–2534, July 2020. ISSN 0962-1083. doi: 10.1111/mec.15507. URL https://onlinelibrary.wiley.com/doi/abs/10.1111/mec.15507. 32542933 PMC7496323

[5] Broder A.Z.. On the resemblance and containment of documents. In Proceedings. Compression and Complexity of SEQUENCES 1997 (Cat. No.97TB100171), pages 21–29. IEEE Comput. Soc. ISBN 0-8186-8132-2. doi: 10.1109/SEQUEN.1997.666900. URL http://ieeexplore.ieee.org/document/666900/.

[6] Bushnell Brian, Rood Jonathan, and Singer Esther. BBMerge – Accurate paired shotgun read merging via overlap. PLOS ONE, 12(10):1–15, 2017. doi: 10.1371/journal.pone.0185056. URL https://doi.org/10.1371/journal.pone.0185056. PMC5657622 29073143

[7] Cay Selahattin Baris, Ulas Cinar Yusuf, Kuralay Selim Can, Inal Behcet, Zararsiz Gokmen, Ciftci Almila, Mollman Rachel, Obut Onur, Eldem Vahap, Bakir Yakup, and Erol Osman. Genome skimming approach reveals the gene arrangements in the chloroplast genomes of the highly endangered Crocus L. species: Crocus istanbulensis (B.Mathew) Rukšāns. PLOS ONE, 17(6):e0269747, June 2022. ISSN 1932-6203. doi: 10.1371/journal.pone.0269747. URL https://dx.plos.org/10.1371/journal.pone.0269747. 35704623 PMC9200356

[8] Ceballos Gerardo, Ehrlich Paul R., Barnosky Anthony D., García Andrés, Pringle Robert M., and Palmer Todd M.. Accelerated modern human–induced species losses: Entering the sixth mass extinction. Science Advances, 1(5):e1400253, June 2015. ISSN 2375-2548. doi: 10.1126/sciadv.1400253. URL https://www.science.org/doi/10.1126/sciadv.1400253. 26601195 PMC4640606

[9] Cerca José, Díaz Patricia Jaramillo, Goubert Clément, Yang Heidi, Bieker Vanessa C., Fernández-Mazuecos Mario, Vargas Pablo, Schley Rowan, Li Siyu, Guevara-Andino Juan Ernesto, Petersen Bent, Petersen Gitte, Sinha Neelima R., Nielsen Lene R., Leebens-Mack James H., Rivas-Torres Gonzalo, Rieseberg Loren H., and Martin Michael D.. Genomic stability in the Galápagos *Scalesia* adaptive radiation: Consistent transposable element accumulation despite hybridization and ecological niche shifts, October 2024. URL http://biorxiv.org/lookup/doi/10.1101/2024.09.30.614436. 10.1186/s13100-025-00362-z PMC12125827 40450335

[10] Chafin Tyler K., Regmi Binod, Douglas Marlis R., Edds David R., Wangchuk Karma, Dorji Sonam, Norbu Pema, Norbu Sangay, Changlu Changlu, Khanal Gopal Prasad, Tshering Singye, and Douglas Michael E.. Parallel introgression, not recurrent emergence, explains apparent elevational ecotypes of polyploid Himalayan snowtrout. Royal Society Open Science, 8(10):210727, October 2021. ISSN 2054-5703. doi: 10.1098/rsos.210727. URL https://royalsocietypublishing.org/doi/10.1098/rsos.210727. 34729207 PMC8548808

[11] Charvel Eduardo, Thomas Isaac, Alves Monteiro Homère J., Sarmashghi Shahab, Dunshea Glenn, Bafna Vineet, and Mirarab Siavash. ReSkmer: modeling repeats allows k-mer-based alignment-free methods to calculate population genomic distances. Genome Biology, May 2026. ISSN 1474-760X. doi: 10.1186/s13059-026-04108-9. URL https://link.springer.com/10.1186/s13059-026-04108-9. PMC13386924 42177542

[12] Coissac Eric, Hollingsworth Peter M., Lavergne Sébastien, and Taberlet Pierre. From barcodes to genomes: extending the concept of DNA barcoding. Molecular Ecology, 25(7):1423–1428, April 2016. ISSN 09621083. doi: 10.1111/mec.13549. URL http://doi.wiley.com/10.1111/mec.13549. ISBN: 0962-1083. 26821259

[13] Duan Han-Ning, Jiang Yin-Zi, Yang Jun-Bo, Cai Jie, Zhao Jian-Li, Li Lu, and Yu Xiang-Qin. Skmer approach improves species discrimination in taxonomically problematic genus Schima (Theaceae). Plant Diversity, 46(6):713–722, November 2024. ISSN 24682659. doi: 10.1016/j.pld.2024.06.003. URL https://linkinghub.elsevier.com/retrieve/pii/S2468265924000945. 39811811 PMC11726044

[14] Fan Huan, Ives Anthony R., Surget-Groba Yann, and Cannon Charles H.. An assembly and alignment-free method of phylogeny reconstruction from next-generation sequencing data. BMC Genomics, 16(1):522, December 2015. ISSN 1471-2164. doi: 10.1186/s12864-015-1647-5. URL http://www.biomedcentral.com/1471-2164/16/522. 26169061 PMC4501066

[15] Huang Weichun, Li Leping, Myers Jason R., and Marth Gabor T.. ART: a next-generation sequencing read simulator. Bioinformatics, 28(4):593–594, 2 2012. ISSN 1460-2059. doi: 10.1093/bioinformatics/btr708. URL https://academic.oup.com/bioinformatics/article-lookup/doi/10.1093/bioinformatics/btr708. 22199392 PMC3278762

[16] Randall Hughes A., Inouye Brian D., Johnson Marc T. J., Underwood Nora, and Vellend Mark. Ecological consequences of genetic diversity. Ecology Letters, 11(6):609–623, June 2008. ISSN 1461-023X, 1461-0248. doi: 10.1111/j.1461-0248.2008.01179.x. URL https://onlinelibrary.wiley.com/doi/10.1111/j.1461-0248.2008.01179.x. 18400018

[17] IPBES. Global assessment report on biodiversity and ecosystem services of the intergovernmental Science-Policy platform on biodiversity and ecosystem services, 2019.

[18] Jain Chirag, Rodriguez-R Luis M, Phillippy Adam M, Konstantinidis Konstantinos T, and Aluru Srinivas. High throughput ANI analysis of 90K prokaryotic genomes reveals clear species boundaries. Nature Communications, 9(1):5114, December 2018. ISSN 2041-1723. doi: 10.1038/s41467-018-07641-9. URL http://www.nature.com/articles/s41467-018-07641-9. arXiv:: http://dx.doi.org/10.1101/225342. PMC6269478 30504855

[19] Jenike Katharine M., Campos-Domínguez Lucía, Boddé Marilou, Cerca José, Hodson Christina N., Schatz Michael C., and Jaron Kamil S.. *k*-mer approaches for biodiversity genomics. Genome Research, page genome;gr.279452.124v1, January 2025. ISSN 1088-9051, 1549-5469. doi: 10.1101/gr.279452.124. URL http://genome.cshlp.org/lookup/doi/10.1101/gr.279452.124. PMC11874746 39890468

[20] Kingman J F C. On the genealogy of large populations. Journal of Applied Probability, 19(1982):27–43, 1982. ISSN 00219002. URL http://www.jstor.org/stable/3213548.

[21] Korneliussen Thorfinn Sand, Albrechtsen Anders, and Nielsen Rasmus. ANGSD: Analysis of next generation sequencing data. BMC Bioinformatics, 15(1):356, November 2014. 25420514 10.1186/s12859-014-0356-4 PMC4248462

[22] Koslicki David and Zabeti Hooman. Improving MinHash via the containment index with applications to metagenomic analysis. Applied Mathematics and Computation, 354:206–215, August 2019. ISSN 00963003. doi: 10.1016/j.amc.2019.02.018. URL https://linkinghub.elsevier.com/retrieve/pii/S009630031930116X.

[23] Li Heng. Aligning sequence reads, clone sequences and assembly contigs with bwa-mem. arXiv preprint arXiv:1303.3997, 2013.

[24] Liu Yanlei, Chen Kai, Wang Lihu, Yu Xinqiang, Xu Chao, Suo Zhili, Zhou Shiliang, Shi Shuo, and Dong Wenpan. Assembly-free reads accurate identification (AFRAID) approach outperforms other methods of DNA barcoding in the walnut family (Juglandaceae). Plant Diversity, 47(1):115–126, January 2025. ISSN 24682659. doi: 10.1016/j.pld.2024.10.002. URL https://linkinghub.elsevier.com/retrieve/pii/S2468265924001677. 40041568 PMC11873577

[25] Marcus Jeffrey M.. Our love-hate relationship with DNA barcodes, the Y2K problem, and the search for next generation barcodes. AIMS Genetics, 5(1):1–23, 2018. ISSN 2377-1143. doi: 10.3934/genet.2018.1.1. URL http://www.aimspress.com/article/10.3934/genet.2018.1.1. 31435510 PMC6690253

[26] Melendez Daira, Berk Sapci Ali Osman, Bafna Vineet, and Mirarab Siavash. SPrUCE: Utilizing ultraconserved elements of DNA for population-level genetic diversity estimation. November 2025. 10.1111/1755-0998.70145 PMC13106921 42026820

[27] Mirarab Siavash and Bafna Vineet. Analyses of Nuclear Reads Obtained Using Genome Skimming. In DeSalle Robert, editor, DNA Barcoding, volume 2744, pages 247–265. Springer US, New York, NY, 2024. ISBN 978-1-0716-3580-3 978-1-0716-3581-0. doi: 10.1007/978-1-0716-3581-0_16. URL https://link.springer.com/10.1007/978-1-0716-3581-0_16. Series Title: Methods in Molecular Biology. 38683324

[28] Mo Zhi-Qiong, Wang Jie, Möller Michael, Yang Jun-Bo, and Gao Lian-Ming. Phylogenetic Relationships and Next-Generation Barcodes in the Genus Torreya Reveal a High Proportion of Misidentified Cultivated Plants. International Journal of Molecular Sciences, 24(17):13216, August 2023. ISSN 1422-0067. doi: 10.3390/ijms241713216. URL https://www.mdpi.com/1422-0067/24/17/13216. 37686021 PMC10487542

[29] Nei M and Li WH. Mathematical model for studying genetic variation in terms of restriction endonucleases. Proceedings of the National Academy of Sciences, 76(10):5269–5273, October 1979. ISSN 0027-8424, 1091-6490. doi: 10.1073/pnas.76.10.5269. URL https://pnas.org/doi/full/10.1073/pnas.76.10.5269. PMC413122 291943

[30] Nei Masatoshi. Analysis of gene diversity in subdivided populations. Proceedings of the national academy of sciences, 70(12):3321–3323, 1973. 10.1073/pnas.70.12.3321 PMC427228 4519626

[31] Nielsen Rasmus and Wakeley John. Distinguishing Migration From Isolation: A Markov Chain Monte Carlo Approach. Genetics, 158(2):885–896, June 2001. ISSN 1943-2631. doi: 10.1093/genetics/158.2.885. URL https://academic.oup.com/genetics/article/158/2/885/6049614. 11404349 PMC1461674

[32] Nishant Koodali T, Singh Nadia D, and Alani Eric. Genomic mutation rates: what high-throughput methods can tell us. Bioessays, 31(9):912–920, 2009. 19644920 10.1002/bies.200900017 PMC2952423

[33] Oksanen Jari, Simpson Gavin L., Blanchet F. Guillaume, Kindt Roeland, Legendre Pierre, Minchin Peter R., O’Hara R.B., Solymos Peter, Henry M. Stevens H., Szoecs Eduard, Wagner Helene, Barbour Matt, Bedward Michael, Bolker Ben, Borcard Daniel, Borman Tuomas, Carvalho Gustavo, Chirico Michael, Caceres Miquel De, Durand Sebastien, Antoniazi Evangelista Heloisa Beatriz, FitzJohn Rich, Friendly Michael, Furneaux Brendan, Hannigan Geoffrey, Hill Mark O., Lahti Leo, Martino Cameron, McGlinn Dan, Ouellette Marie-Helene, Ribeiro Cunha Eduardo, Smith Tyler, Stier Adrian, Ter Braak Cajo J.F., and Weedon James. vegan: Community Ecology Package, 2026. URL https://vegandevs.github.io/vegan/. R package version 2.8-0.

[34] Ondov Brian D, Treangen Todd J, Melsted Páll, Mallonee Adam B, Bergman Nicholas H, Koren Sergey, and Phillippy Adam M. Mash: fast genome and metagenome distance estimation using MinHash. Genome Biology, 17(1):132, December 2016. ISSN 1474-760X. doi: 10.1186/s13059-016-0997-x. 27323842 PMC4915045

[35] Pereira Henrique Miguel, Ferrier Simon, Walters Michele, Geller Gary N, Jongman Rob HG, Scholes Robert J, Bruford Michael William, Brummitt Neil, Butchart Stuart HM, Cardoso AC, Essential biodiversity variables. Science, 339(6117):277–278, 2013. 23329036 10.1126/science.1229931

[36] Tessa Pierce N., Irber Luiz, Reiter Taylor, Brooks Phillip, and Brown C. Titus. Large-scale sequence comparisons with sourmash. F1000Research, 8:1006, July 2019. ISSN 2046-1402. doi: 10.12688/f1000research.19675.1. URL https://f1000research.com/articles/8-1006/v1. 31508216 PMC6720031

[37] Quicke Donald L. J., Smith M. Alex, Janzen Daniel H., Hallwachs Winnie, Fernandez-Triana Jose, Laurenne Nina M., Zaldívar-Riverón Alejandro, Shaw Mark R., Broad Gavin R., Klopfstein Seraina, Shaw Scott R., Hrcek Jan, Hebert Paul D. N., Miller Scott E., Rodriguez Josephine J., Whitfield James B., Sharkey Michael J., Sharanowski Barbara J., Jussila Reijo, Gauld Ian D., Chesters Douglas, and Vogler Alfried P.. Utility of the DNA barcoding gene fragment for parasitic wasp phylogeny (Hymenoptera: Ichneumonoidea): data release and new measure of taxonomic congruence. Molecular Ecology Resources, 12(4):676–685, July 2012. ISSN 1755-098X, 1755-0998. doi: 10.1111/j.1755-0998.2012.03143.x. URL https://onlinelibrary.wiley.com/doi/10.1111/j.1755-0998.2012.03143.x. 22487608

[38] Rachtman Eleonora, Balaban Metin, Bafna Vineet, and Mirarab Siavash. The impact of contaminants on the accuracy of genome skimming and the effectiveness of exclusion read filters. Molecular Ecology Resources, 20(3):1755–0998.13135, May 2020. ISSN 1755-098X. doi: 10.1111/1755-0998.13135. URL https://onlinelibrary.wiley.com/doi/abs/10.1111/1755-0998.13135. 31943790

[39] Rachtman Eleonora, Sarmashghi Shahab, Bafna Vineet, and Mirarab Siavash. Quantifying the uncertainty of assembly-free genome-wide distance estimates and phylogenetic relationships using subsampling. Cell Systems, 13(10):817–829.e3, October 2022. ISSN 24054712. doi: 10.1016/j.cels.2022.06.007. URL https://linkinghub.elsevier.com/retrieve/pii/S2405471222002770. 36265468 PMC9589918

[40] Rahman Hera Mahmudur and Koslicki David. Estimating similarity and distance using FracMinHash. Algorithms for Molecular Biology, 20(1):8, May 2025. ISSN 1748-7188. doi: 10.1186/s13015-025-00276-8. URL https://almob.biomedcentral.com/articles/10.1186/s13015-025-00276-8. 40375084 PMC12082993

[41] Reed David H. and Frankham Richard. Correlation between Fitness and Genetic Diversity. Conservation Biology, 17(1):230–237, February 2003. ISSN 0888-8892, 1523-1739. doi: 10.1046/j.1523-1739.2003.01236.x. URL https://conbio.onlinelibrary.wiley.com/doi/10.1046/j.1523-1739.2003.01236.x.

[42] Reynolds John, Weir Bruce S, and Cockerham C Clark. Estimation of the coancestry coefficient: basis for a short-term genetic distance. Genetics, 105(3):767–779, 1983. 17246175 10.1093/genetics/105.3.767 PMC1202185

[43] Sarmashghi Shahab, Bohmann Kristine, Gilbert M. Thomas P., Bafna Vineet, and Mirarab Siavash. Skmer: assembly-free and alignment-free sample identification using genome skims. Genome Biology, 20 (1):34, December 2019. ISSN 1474-760X. doi: 10.1186/s13059-019-1632-4. URL https://genomebiology.biomedcentral.com/articles/10.1186/s13059-019-1632-4. 30760303 PMC6374904

[44] Sarmashghi Shahab, Balaban Metin, Rachtman Eleonora, Touri Behrouz, Mirarab Siavash, and Bafna Vineet. Estimating repeat spectra and genome length from low-coverage genome skims with RESPECT. PLOS Computational Biology, 17(11):e1009449, November 2021. ISSN 1553-7358. doi: 10.1371/journal.pcbi.1009449. URL https://dx.plos.org/10.1371/journal.pcbi.1009449. 34780468 PMC8629397

[45] Shaw Jim and Yu Yun William. Fast and robust metagenomic sequence comparison through sparse chaining with skani. Nature Methods, September 2023. ISSN 1548-7091, 1548-7105. doi: 10.1038/s41592-023-02018-3. URL https://www.nature.com/articles/s41592-023-02018-3. PMC10630134 37735570

[46] Slatkin Montgomery. Isolation by distance in equilibrium and non-equilibrium populations. Evolution, 47(1):264–279, 1993. 28568097 10.1111/j.1558-5646.1993.tb01215.x

[47] Tang Kujin, Ren Jie, and Sun Fengzhu. Afann: bias adjustment for alignment-free sequence comparison based on sequencing data using neural network regression. Genome Biology, 20(1):266, December 2019. ISSN 1474-760X. doi: 10.1186/s13059-019-1872-3. URL https://genomebiology.biomedcentral.com/articles/10.1186/s13059-019-1872-3. 31801606 PMC6891986

[48] Weir Bruce S and Cockerham C Clark. Estimating f-statistics for the analysis of population structure. evolution, pages 1358–1370, 1984. 28563791 10.1111/j.1558-5646.1984.tb05657.x

[49] Wilkinson-Herbots Hilde M.. Genealogy and subpopulation differentiation under various models of population structure. Journal of Mathematical Biology, 37(6):535–585, December 1998. ISSN 0303-6812, 1432-1416. doi: 10.1007/s002850050140. URL http://link.springer.com/10.1007/s002850050140.

[50] Wood Derrick E., Lu Jennifer, and Langmead Ben. Improved metagenomic analysis with Kraken 2. Genome Biology, 20(1):257, December 2019. ISSN 1474-760X. doi: 10.1186/s13059-019-1891-0. URL https://genomebiology.biomedcentral.com/articles/10.1186/s13059-019-1891-0. 31779668 PMC6883579

[51] Xu Shengyong, Xiao Shijun, Zhu Shilin, Zeng Xiaofei, Luo Jing, Liu Jiaqi, Gao Tianxiang, and Chen Nansheng. A draft genome assembly of the chinese sillago (sillago sinica), the first reference genome for sillaginidae fishes. GigaScience, 7(9):giy108, 2018. 30202912 10.1093/gigascience/giy108 PMC6143730

[52] Zhang Le, Huang Yi-Wei, Huang Jia-Lin, Ya Ji-Dong, Zhe Meng-Qing, Zeng Chun-Xia, Zhang Zhi-Rong, Zhang Shi-Bao, Li De-Zhu, Li Hong-Tao, and Yang Jun-Bo. DNA barcoding of Cymbidium by genome skimming: Call for next-generation nuclear barcodes. Molecular Ecology Resources, 23(2): 424–439, February 2023. ISSN 1755-098X, 1755-0998. doi: 10.1111/1755-0998.13719. URL https://onlinelibrary.wiley.com/doi/10.1111/1755-0998.13719. 36219539

[53] Zhao Xiang, Zheng Tianlun, Gao Tianxiang, and Song Na. Whole-genome resequencing reveals genetic diversity and selection signals in warm temperate and subtropical sillago sinica populations. BMC genomics, 24(1):547, 2023. 37715145 10.1186/s12864-023-09652-3 PMC10503073

[54] Berk Şapcı Ali Osman, Rachtman Eleonora, and Mirarab Siavash. CONSULT-II: Accurate taxonomic identification and profiling using locality-sensitive hashing. Bioinformatics, page btae150, March 2024. ISSN 1367-4811. doi: 10.1093/bioinformatics/btae150. URL https://academic.oup.com/bioinformatics/advance-article/doi/10.1093/bioinformatics/btae150/7630488. 38492564 PMC10985673

